# Chilling-induced DNA Demethylation is associated with the cold tolerance of *Hevea brasiliensis*

**DOI:** 10.1186/s12870-018-1276-7

**Published:** 2018-04-23

**Authors:** Xiao Tang, Qichao Wang, Hongmei Yuan, Xi Huang

**Affiliations:** Hainan Key Laboratory for Sustainable Utilization of Tropical Bioresources, Institute of Tropical Agriculture and Forestry, Hainan University, Renmin Rd. 58, Haikou, 570228 People’s Republic of China

**Keywords:** *Hevea brasiliensis*, Cold tolerance, DNA demethylation, *HbICE1*, *HbMET*

## Abstract

**Background:**

Low temperature influences the development and latex production of rubber trees (*Hevea brasiliensis*) when extension to suboptimal high-latitude areas. The successful extension of *Hevea brasiliensis* cultivation to high-latitude areas has long believed to benefit from the breeding of cold-tolerant cultivars. A puzzling incongruity is the variation in cold tolerance among the cultivated clones despite their similar genetic make-up.

**Results:**

To investigate this, we first transferred cultivar Reyan 7–33-97 to short-term cold treatment, and showed that cold-related genes (such as *HbICE1* and *HbCBF2*), cold-responsive (*COR*) genes, and DNA-methylation related genes (such as *HbMET1*) were induced by cold treatment. Furthermore, long-term cold treatment not only elevated the transcriptional activities of the *HbICE1*, *HbCBF2*, and *HbMET*, but also induced DNA demethylation of their promoters. Cold treatment increased the transcriptional activities of demethylation-related genes such as the *HbDME*, *HbROS*, and *HbDML* genes, but did not alter the promoter methylation status. Furthermore, the *HbICE1* and *HbMET* promoters showed hypomethylation status in samples collected at the end of winter from 12 different cultivars grown in four geographical locations, but switched to hypermethylation status at the end of summer. Expression of *COR* was correlated with the low temperature. Given that little genetic diversity exists in the *HbICE1* and *HbMET* promoters among different cultivars, the DNA demethylation induced by cold was highly correlated with low temperature, but not with the genetic backgrounds of cultivars.

**Conclusion:**

Cold-induced epigenetic modification might play an important role in cold tolerance of *H. brasiliensis.*

**Electronic supplementary material:**

The online version of this article (10.1186/s12870-018-1276-7) contains supplementary material, which is available to authorized users.

## Background

*Hevea brasiliensis* Muell. Arg. is the major source of commercial natural rubber and is native to the rainforests of tropical regions of the Amazonian basin. In 1876, Henry Wickham collected 70,000 seeds of rubber tree in Brazil and smuggled to England. The seedlings, known as ‘Wickham base’, were later sent to Southeast Asian countries and became the major germplasm for selection of rubber cultivars [1]. In the late 1970s, rubber plantations spread to sub-optimal environments worldwide; e.g. southern China, southern Brazil, highland areas of Vietnam, and northeast India. These areas have diverse climates and environmental stresses, such as low temperature, high latitude, disease, drought, and so on. A number of rubber clones were developed to exhibit specific adaptation to these areas. While RRIM 600 is a universally adapted clone in these sub-optimal areas, the RRII 208, RRII 203, PB 235, and Haiken 1 (India); GT 1 (Vietnam); Reyan 7–33-97 and PB 235 (China); and FX-3864 and PB 235 (Brazil) clones exhibit specific adaptations for yield in different countries [2]. Low temperature influences not only the latex production but the development of rubber trees and limits development of rubber plantations in high-latitude areas. Lower temperatures are also responsible for stopping rubber production for 2–3 months per year in sub-optimal areas [3, 4]. Therefore, breeding clones with enhanced cold tolerance is of great significance.

Low temperature is one of the most limiting factors for plants. Plants adapt to seasonal temperature oscillations by adjusting their metabolism and changing the content of a range of cryo-protective compounds to enhance their cold tolerance. Major step to elucidate the signalling networks of cold-stress response is characterization of C-repeat-binding factors (CBFs) and dehydration-responsive element-binding factors 1 (DREB1s) [5–8]. Inducer of CBF expression 1 (ICE1) and calmodulin binding transcription activator 3 (CAMTA3) positively regulates the expression of *CBF* genes [5, 9]. Whereas MYB15 and ethylene insensitive 3 (EIN3) are negative regulators of CBF genes [10, 11], brassinazole-resistant 1 (BZR1) positively regulates freezing tolerance by acting upstream of *CBF1* and *CBF2* to directly regulate their expression [12]. Most recently, phytochrome-interacting factor 3 (PIF3) has been shown to function as a negative regulator of *Arabidopsis* freezing tolerance by directly binding to the promoters of CBF genes to inhibit their expression [13]. Besides transcriptional regulation, several factors regulate ICE-CBF pathway at posttranslational levels. For example, SIZ1 (SAP and Miz1) was reported to mediate sumoylation of ICE1 and up-regulate CBF3 expression [14]. In contrast, RING E3 ligase, HOS1 (high expression of osmotically responsive genes1) negatively regulate plant cold responses via mediating the ubiquitination and degradation of ICE1 [15]. A recently identified kinase, open stomata 1 (OST1), phosphorylated and stabilized ICE1, and promoted the expression of CBF expression and freezing tolerance [16]. Other protein kinases, such as mitogen-activated protein kinases MPK3 and MPK6, have been implicated in phosphorylate the ICE1 protein to negatively regulates ICE1 stability and freezing tolerance in *Arabidopsis* [17]. Liu et al. demonstrated that the cold-activated plasma membrane CRPK1 phosphorylates 14–3-3 proteins, which are imported from the cytosol to the nucleus promote the degradation of CBFs via the 26S proteasome pathway [18]. The *ICE-CBF* transcriptional cascade was proposed to play a central role in cold responses of plants [19].

A growing body of evidence reveals that epigenetic modifications (e.g. DNA methylation and histone modifications) play an important role in regulation of stress-responsive genes in plants [20–23]. Epigenetic mechanisms can bring about heritable phenotypic changes by regulating gene expression without changing DNA sequences [24, 25]. DNA cytosine methylation is an evolutionarily conserved chromatin modification that contributes to gene regulation and genome structure and integrity [26]. In plants, cytosine methylation occurs at CG, CHG, and CHH sites (where H represents A, C, or T) [27, 28]. Plants encode diverse DNA methyltransferases that specially target cytosines for methylation in specific DNA sequence contexts, including DOMAINS REARRANGED METHYLTRANSFERASE 2 (DRM2), the CHROMOMETHYLASES (CMTs), and METHYLTRANSFERASE 1 (MET1) [29]. Cytosine methylation is established and maintained by METs using S-adenosyl-L-methionine (SAM) as the methyl donor. Maintenance of DNA methylation in CG and CHG contexts is mediated by MET1 and CMT3, respectively [27]. CMT2 in Arabidopsis was found to be associated with differential CHH methylation in transposable elements [30]. CMT3 is required for the establishment of gene body methylation [26]. Cytosine methylation of asymmetric CHH context can’t be maintained and just de novo mediated by DRM2 protein during every cell cycle [31, 32]. Pre-existing DNA methylation can be lost in passive or active demethylation processes. Passive demethylation occurs when failure to maintain DNA methylation owing to a shortage of methyl groups or dysfunction of DNA methyltransferase, whereas active removal of cytosine methylation is catalysed by the DNA glycosylase family, such as demeter (DME) and their paralogs demeter-like (DML2 and DML3) proteins, and repressor of silencing 1 (ROS1) [27, 33–35].

Expression of many epigenetic regulators is regulated by low temperature. In Arabidopsis, HDA6 has been identified as a putative histone deacetylase needed to enhance DNA methylation induced by double-stranded RNA [36]. *HDA6* is induced by long-term cold treatment, and regulates locus-directed heterochromatin silencing in cooperation with MET1, possibly recruiting MET1 to specific loci [37]. RDM4 (RNA-directed DNA methylation 4) modulates cold-stress resistance through the CBF-mediated pathway in *Arabidopsis* [20]. Expression of *HDAC* genes was increased after cold acclimation, and global deacetylation of histone (H3 and H4) was observed in maize [38]. Moreover, tandem repeats in heterochromatin were selectively unsilenced under cold stress, which is associated with H3K9ac accumulation and reduction of DNA methylation and H3K9me2 [39]. Cold-induced epigenetic changes result in the expression of *COR* genes, contributing to stress tolerance [40]. Vernalisation is the best-characterised pathway involved in cold induced epigenetic regulation [41–43]. However, our understanding of the epigenetic regulation underlying cold acclimation is limited [40].

Due to the extension of rubber cultivation to sub-optimal areas, well beyond the native environment of 10°N/S of the equator and 400 m above sea level, the cold tolerance of *H. brasiliensis* has been a focus of research. Several studies investigated altered physiological parameters under cold stress [4, 44, 45], but the molecular mechanisms of cold responses in *H. brasiliensis* have not been systemically investigated. In this work, we analyse the expression profiles and DNA methylation patterns of cold- and methylation-related genes in *H. brasiliensis* clones from different geographical locations. Our results suggest that cold-induced epigenetic modifications play a more important role than genetic variation in cold acclimation of *H. brasiliensis*.

## Methods

### Plant materials and treatments

Sixty-day-old seedlings of *H. brasiliensis* cultivar Reyan 7–33-97 self-rooting juvenile clones were provided by the Chinese Academy of Tropical Agriculture Sciences (Danzhou Hainan, P.R. China). The seedlings were cultivated in a growth chamber (MLR-352H-PC, Panasonic, Japan) under the control conditions: 16-h light (100 lx) /8-h dark, 75% relative humidity, at 28 °C. For cold treatment, the temperature of the growth chamber was switched to 19 °C or 4 °C while the setting for lighting, photoperiod and relative humidity was kept intact. For short-term cold treatment, leaf samples were collected at 0, 3, 6, 12, and 24 h after treatment. For long-term cold treatment, the seedlings were cultivated at 19 °C for 1 month (1-CT), transferred to 28 °C for recovery for 1 month, then transferred to 19 °C for 1 month (2-CT). After recovery for 1 month, the seedlings were cultivated at 19 °C for 1 month (3-CT). After each treatment, the treated and control seedlings (cultivated at 28 °C [mock]) were photographed. Leaf samples were collected and immediately frozen, ground in liquid nitrogen, and stored at − 80 °C until RNA and DNA extraction. Five seedlings were selected for each treatment and three independent biological replicates were performed.

Leaf samples from four different geographical locations were collected from mature rubber trees in April and October 2016. Samples of cultivars Yunyan77–4, Yunyan77–2, GT1, RRIM600, and Reyan87–3 were collected from an experimental plantation of the College of Tropical Crop Science, Yunnan Agricultural University in Puer, Yunnan Province. Samples of cultivars Yunyan77–4, Yunyan77–2, Yunyan73–46, PR107, and Reyan 87–9 were collected from an experimental plantation of the Yunnan Institute of Tropical Crops in Xishuangbanna, Yunnan Province. Samples of cultivars Wenchang11, Reyan88–13, Reyan7–18-55, RRIM600, and PR107 were collected from an experimental plantation of the Wenchang Rubber Research Institute in Wenchang, Hainan Province. Samples of RRIM600, PR107, and Reyan7–18-55 were collected from Nanxin farm in Sanya, Hainan Province. The mean temperature, longitude, and latitude of the four locations are listed in Table 1. Leaf samples were collected from five individual trees of each cultivar per site, wrapped in aluminium foil and frozen in liquid nitrogen. Three independent biological replicates were performed. The samples were ground in liquid nitrogen and stored at − 80 °C until RNA and DNA extraction. Genomic DNA was isolated according to a method described previously [46]. Total RNA was extracted as described elsewhere [47].

**Table 1:** Locations of the rubber plantations selected for leaf sample collection

Location	Latitude	Longitude	Mean temperature^a^		
			Annual	Summer.	Winter
Sanya	N18°19′58.58′′	E109°27′35.35′′	25.7 °C	28.7 °C	21.4 °C
Wenchang	N19°37′37.29′′	E110°38′37.29′′	24.4 °C	28.3 °C	19.3 °C
Xishuangbanna	N22°00′55.15′′	E100°46′13.36′′	21.8 °C	25.0 °C	16.3 °C
Puer	N22°47′45.75′′	E100°58′57.72′′	19.0 °C	22.3 °C	14.0 °C

^a^Temperatures are from the China Meteorological Administration (www.cma.gov.cn)

### Quantitative real-time PCR (qRT-PCR)

First-strand cDNA was synthesised using a cDNA synthesis kit according to the manufacturer’s instructions (Fermentas, Vilnius, Lithuania). qRT-PCR was performed according to Wang’s method [48]. The primer sets for qRT-PCR are listed in Additional file 1: Table S1. *HbRH8* is used as internal control for expression profile in different tissue suggested by Li et al., [49]. *HbACT7b* was used as control for other qRT-PCR. Relative expression levels were calculated by the 2^–ΔΔCT^ method. Each biological sample was performed with three independent repetitions [50]. Statistical analysis was performed by Duncan’s multiple range test with SPSS software. Means were considered significantly different at values of *P* < 0.05 and < 0.01. Cluster analysis of transcriptional profiles was performed using CLUSTER software (http://bonsai.hgc.jp/~mdehoon/software/cluster/software.htm). The heatmap was generated by Java Treeview 4 software [51].

### Bioinformatics analysis of promoters

Promoter sequences were predicted using Bioedit software (http://www.mbio.ncsu.edu/BioEdit/bioedit.html) and confirmed using BLASTP at NCBI (https://blast.ncbi.nlm.nih.gov/Blast.cgi). In silico cloning was carried out as described previously [52]. Promoter fragments (200–400 bp) were amplified by PCR using the primers in Additional file 1: Table S2. The *cis*-elements of the promoters were predicted by PlantCare online software (http://bioinformatics.psb.ugent.be/webtools/plantcare/html/)(Additional file 1: Table S3) [53].

### Methylated CpG island screening

Five micrograms of genomic DNA were digested by *Sma*I overnight to cleave unmethylated CCCGGG to generate blunt-end products, then by *Xma*I to cleave CCCGGG irrespective of methylation status to generate cohesive-end products. The 5′-CCGGTAGCTAATGAACCAT-3′ and 5′-ATCGATTACTTGGTA -3′ oligonucleotides (50 μM each) were mixed and annealed at 65 °C for 5 min. A 500-ng aliquot of digested DNA was ligated with T4 ligase (TaKaRa, Dalian) at 16 °C overnight. The ligated fragments were amplified by PCR at 96 °C for 20 s, 58 °C for 25 s, and 72 °C for 2 min for 30 cycles using a single adaptor-specific primer (5′-ATGGTTCATTAGCTACCGGG-3′). Differential display screening was performed by amplifying DNA fragments by PCR using the initial PCR product as a template and a 10-base oligonucleotide primer. PCR products were separated on agarose gel and stained with ethidium bromide [54].

### Bisulphite sequencing

Genomic DNA was isolated and subjected to bisulphite treatment using an EZ DNA Methylation-Gold kit (Zymo Research, Orange, CA, USA) according to the manufacturer’s instructions. Bisulphite-treated and intact DNAs (not treated with bisulphite) were amplified by PCR using the primers in Additional file 1: Table S2. The PCR products were ligated into the pMD19-T vector (TaKaRa, Dalian) and transformed into *E. coli* DH5a strain for bisulphite sequencing. To check the conversion rate of EZ DNA Methylation-Gold kit, plasmid containing unmethylated DNA isolated from *E. coli* was treated with or without bisulphite and used as PCR template. The PCR products were further ligated into pMD19-T vector and transformed into *E. coli* DH5a strain. Ten clones were randomly selected for sequencing. The conversion rate of bisulfite sequencing was calculated. Threshold of the conversion rate to decide “unmethylated” was selected as ≥90%. Methylation patterns were evaluated using CyMATE software. The sequences were aligned using MEGA.6 and ClustalW server (http://www.ch.embnet.org/software/ClustalW.html) and analysed using Cytosine Methylation Analysis Tool for Everyone (CyMATE) software (http://cymate.org/cymate.html) [55].

## Results

### Gene expression profiles after short-term cold treatment

**Fig. 1:**
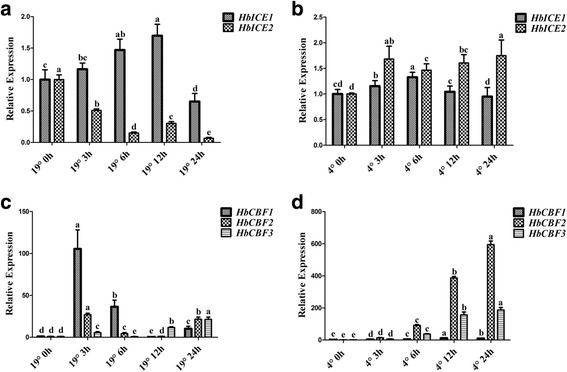
Chilling treatment induced the expression of the *HbICE* and *HbCBF* genes of *H. brasiliensis*. Seedlings of *H. brasiliensis* were treated at 19 °C (**a** and **c**) or 4 °C (**b** and **d**). Samples were collected at the time points indicated. The expression levels of *HbICE* (**a** and **b**) and *HbCBF* (**c** and **d**) genes were analysed by qRT-PCR. Values are presented as the mean standard error of three independent biological replicates. Different letters indicate significant differences (*P* < 0.05) according to one-way analysis of variance

**Fig. 2:**
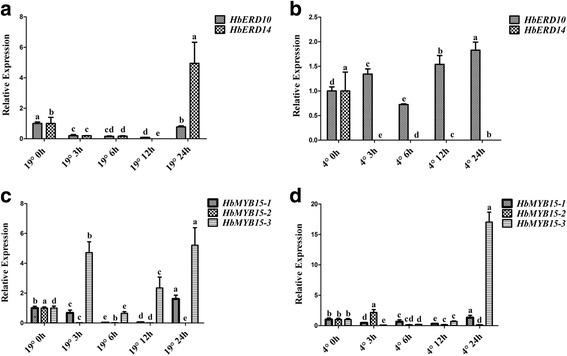
Cold treatment induced the expression of COR genes of *H. brasiliensis*. Seedlings of *H. brasiliensis* were treated at 19 °C (**a** and **c**) or 4 °C (**b** and **d**). Leaf samples were collected at the time points indicated. The expression levels of *HbERD* (**a** and **b**) and *HbMYB15* (**c** and **d**) genes were analysed by qRT-PCR. Values are presented as the mean standard error of three independent biological replicates. Different letters indicate significant differences (*P* < 0.05) according to one-way analysis of variance

To investigate cold-responsive genes of *H. brasiliensis*, 60-day-old seedlings were transferred to 19 °C or treated at 4 °C and gene expression was analysed by qRT-PCR. *HbICE1* expression was induced at 3 h, and continuously up-regulated to 12 h, but down-regulated at 24 h after 19 °C treatment, whereas the expression of *HbICE2* was continuously suppressed from 3 h to 24 h after treatment (Fig. 1a). In contrast, both *HbICE1* and *HbICE2* were upregulated after treatment at 4 °C; the induction of *HbICE2* was more durable and stronger than that of *HbICE1* (Fig. 1b). *HbCBFs* expression showed a similar pattern*. HbCBF1* was strongly induced at 3 h and 6 h and returned to low level at 16 h after 19 °C treatment, whereas *HbCBF2* and *HbCBF3* expression was only slightly increased (Fig. 1c). When treated at 4 °C, *HbCBF2* and *HbCBF3* were continuously upregulated from 3 h to 24 h after treatment, but *HbCBF1* was not (Fig. 1d). Additionally, *COR* genes (including two early response to dehydration genes [*HbERD10* and *HbERD14*] and three *HbMYB* genes) were analysed. After treatment at 19 °C, *HbERD14* and *HbMYB15–3* were upregulated at 24 h and 3 h, respectively (Fig. 2a and c). Under 4 °C, *HbERD14* was sharply down-regulated, whereas *HbERD10* showed durable upregulation and *HbMYB15–3* were upregulated only at 24 h after treatment (Fig. 2b and d). These data suggest that different members of the *ICE/CBF* and *COR* gene families responded to different levels of cold stress and showed different temporal dynamics of expression levels.

**Fig. 3:**
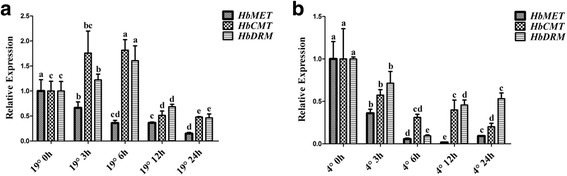
Cold treatment altered the expression levels of DNA methylation related genes in *H. brasiliensis*. Seedlings of *H. brasiliensis* were treated at 19 °C (**a**) or 4 °C (**b**). Leaf samples were collected at the time points indicated. The expression levels of *HbMET*, *HbCMT*, and *HbDRM* were analysed by qRT-PCR. Values are presented as the mean standard error of three independent biological replicates. Different letters indicate significant differences (*P* < 0.05) according to one-way analysis of variance

To determine whether epigenetic modification plays a role in the cold response of *H. brasiliensis*, we assessed the expression of three DNA methylation-related genes. *HbCMT* and *HbDRM* were induced at 3 h and 6 h after treatment at 19 °C, but showed steady suppression at 4 °C, whereas *HbMET1* was continuously downregulated by 19 °C and 4 °C (Fig. 3), suggesting that DNA methylation is involved in the cold response of *H. brasiliensis*.

### Long-term cold treatment influences phenotype and gene expression profiles

**Fig. 4:**
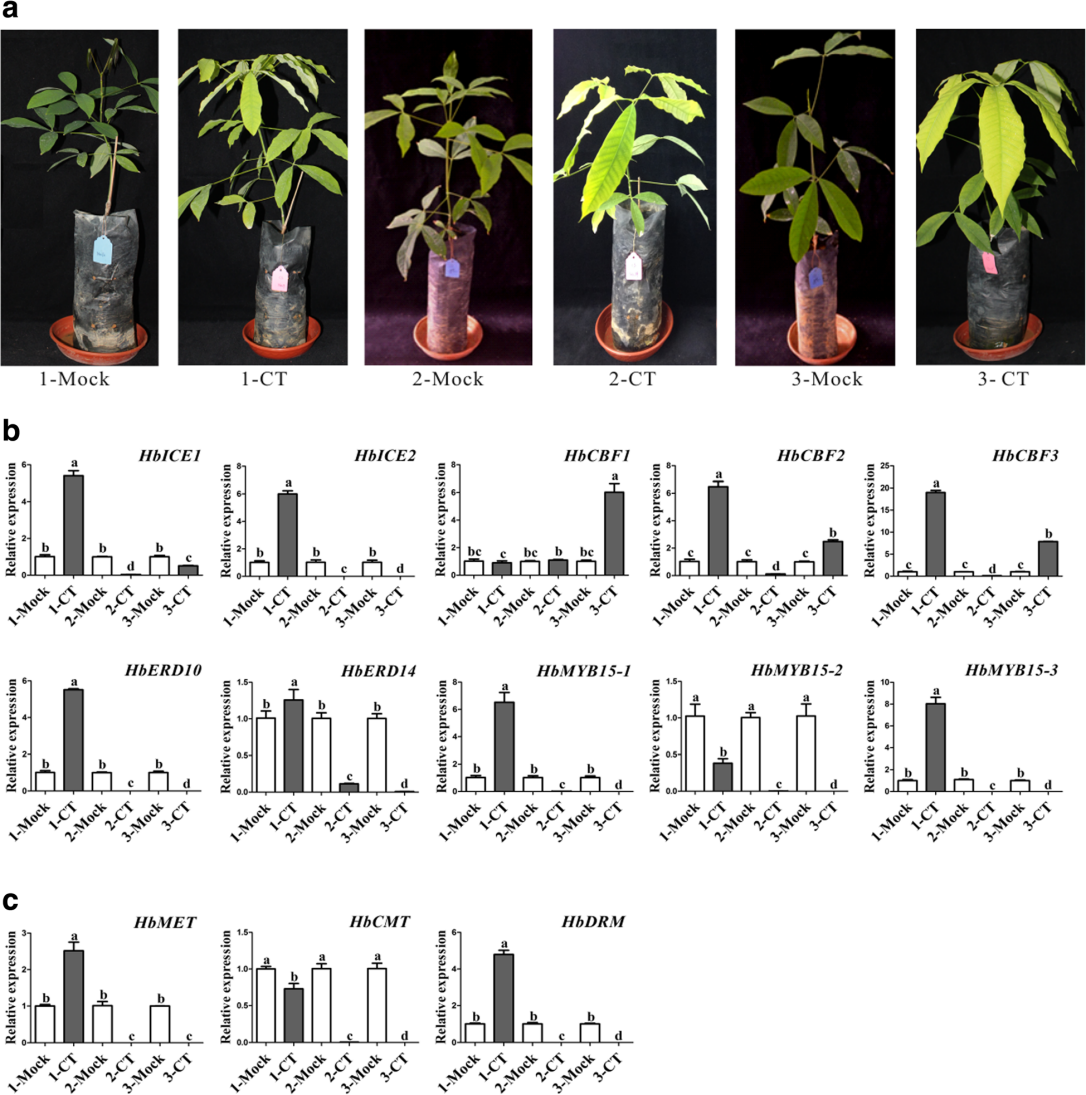
Effect of long-term cold treatment on phenotype and gene expression patterns. Seedlings of *H. brasiliensis* were treated at 19 °C for 1 month (1-CT), transferred to 28 °C for recovery for 1 month, then treated at 19 °C for 1 month (2-CT). After recovery for 1 month, the seedlings were again cold-treated for 1 month (3-CT). After each treatment, the phenotypes were photographed (**a**) and leaf samples were collected. The expression levels of cold-related genes (**b**) and methylation-related genes (**c**) were analysed by qRT-PCR. Values are presented as the mean standard error of three independent biological replicates. Different letters indicate significant differences (*P* < 0.05) according to one-way analysis of variance

To investigate whether long-term cold treatment alters phenotype and the expression of *COR* genes, *H. brasiliensis* seedlings were treated at 19 °C. Because continuous cold treatment might lead to leaf falling, the seedlings were treated at 19 °C for 1 month (1-CT), then transferred to 28 °C for recovery for 1 month, followed by treatment at 19 °C for 1 month (2-CT) or 2 months (3-CT) with a 1-month interval for recovery at 28 °C. As shown in Fig. 4a, the 1-Mock, 2-Mock, and 3-Mock controls (cultivated at 28 °C) did not show evident alteration of phenotype, whereas all cold-treated seedlings (1-CT, 2-CT, and 3-CT) showed leaf chlorosis and growth retardation. Leaf of the mock seedlings was greener than the cold treated leaf, suggesting that development or maintenance of chloroplast might be affected by low temperature. The expression-levels of *HbICE1* and *HbICE2* were much higher in 1-CT than in 1-Mock, but significantly lower in 2-CT and 3-CT than in 2-Mock and 3-Mock. While *HbCBF2* was rapidly induced in 1-CT, *HbCBF1* was just up-regulated in 3-CT compared with each mock control. Similar to the expression profiles of *HbICEs HbMYB15–1*, *HbMYB15–3*, and *HbERD* genes were upregulated in 1-CT and down regulated in 2-CT and 3-CT (Fig. 4b), whereas *HbMYB15–2* was always suppressed in all 1-CT, 2-CT and 3-CT. Interestingly, all three methylation-related genes were responsive to long-term cold treatment. *HbDRM* expression was induced after the first (1-CT) and second (2-CT) cold treatments, but returned to normal after the third cold treatment (3-CT), while *HbMET* and *HbCMT* expression did not increase until the third cold treatment (3-CT) (Fig. 4c), suggesting that long-term cold treatment might alter DNA methylation status in *H. brasiliensis*.

### Long-term cold treatment altered DNA methylation patterns

To determine whether long-term cold treatment altered the genome-wide DNA methylation status, we performed methylated CpG island screening. Genomic DNA was digested with *Sma*I followed by *Xma*I. The DNA fragments were ligated with the adapter sequence and amplified by PCR. The PCR band profiles of the 1-Mock, 2-Mock, and 3-Mock samples were similar. However, the 1-CT, 2-CT, and 3-CT samples showed different band profiles (Additional file 1: Figure S1), suggesting that cold treatment induced significant alteration of the DNA methylation status of CpG islands throughout the genome.

**Fig. 5:**
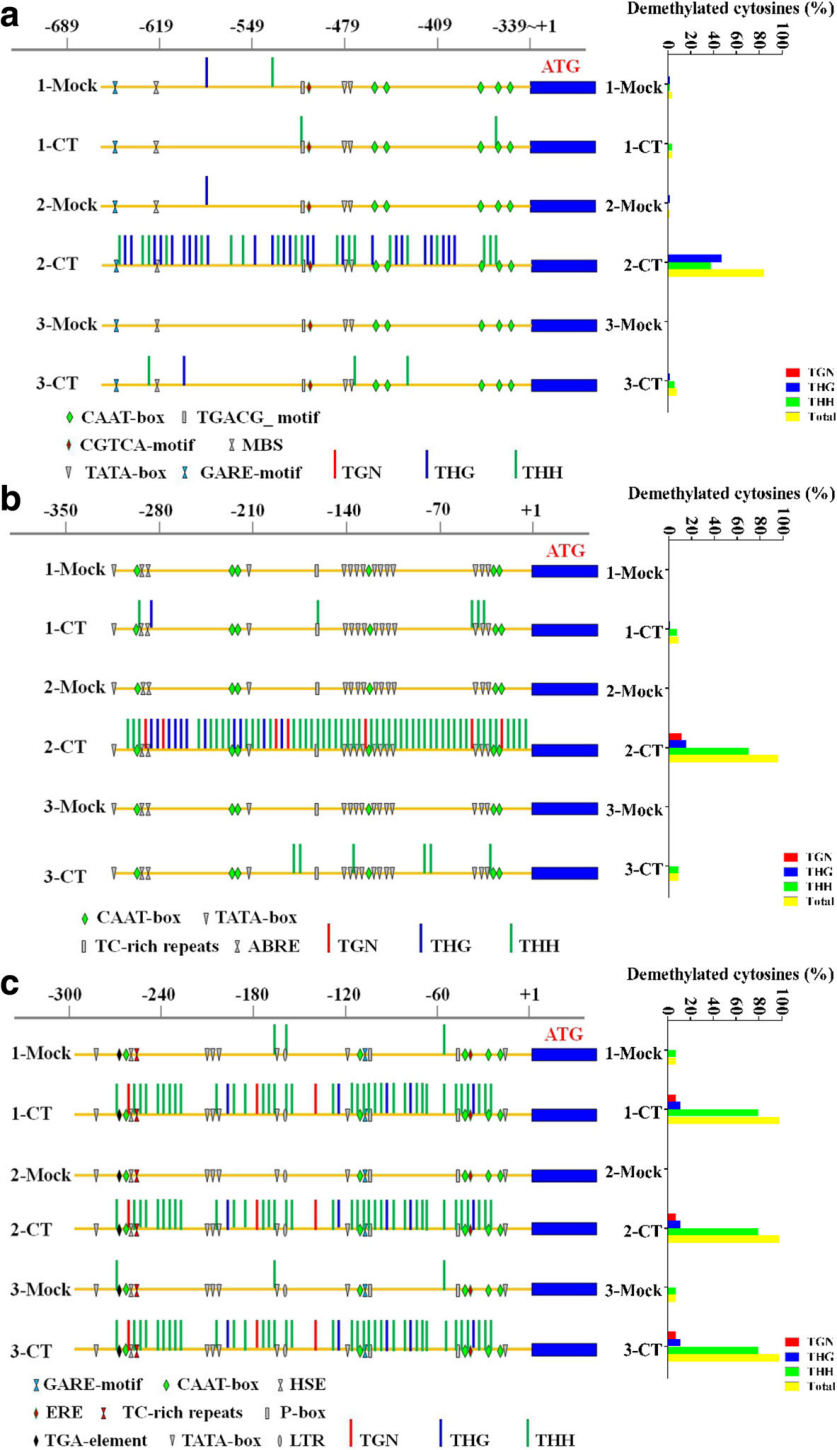
Long-term cold treatment induced demethylation of promoters. Seedlings of *H. brasiliensis* were treated at 19 °C for 1 month (1-CT), transferred to 28 °C for recovery for 1 month, and then treated at 19 °C for 1 month (2-CT). After recovery for 1 month, the seedlings were again cold-treated for 1 month (3-CT). Leaf samples were collected after each treatment. Total DNA was isolated and treated with or without bisulphite. Methylation patterns were evaluated using CyMATE software. Probable TGN, THG, and THH demethylation sites were identified by the software and projected symbolically. The percent of each demethylation type were indicated at the right side. (**a**) A 324-bp region of the *HICE1* promoter. (**b**) A 318-bp region of the *HbCBF2* promoter. (**c**) A 287-bp fragment of the *HbMET* promoter

To determine whether long-term cold treatment altered the DNA methylation pattern of *COR* and methylation-related genes, the promoter sequences of *HbICE1*, *HbCBF2*, and *HbMET* were bisulphite-sequenced. Conversion rates of plasmid DNA of *HbICE1*, *HbCBF2*, and *HbMET* promoters using EZ DNA Methylation-Gold kit were 95.51%, 91.41% and 96.36%, respectively (Additional file 1: Figure S2), suggesting that the unmethylated cytosine could be efficiently conversed by the kit. 90% of the conversion rate was selected as a threshold to decide methylated or not methylated in the following study. The 324-bp sequence of the *HbICE1* promoter contained *cis*-regulatory elements such as ARE, CATT-motif, CAAT-box, CCAAT-box, CGTCA and TGACG-palindrome motif, MBS, TATA-box, and several unnamed motifs (Additional file 1: Table S3). In total, 51 CpG islands were present in the examined fragment, including 2 CGN, 27 CHG, and 21 CHH methylation sites. In the 1-Mock, 2-Mock, and 3-Mock control samples, few methylation sites in the *HbICE1* promoter were altered. After the second cold treatment (2-CT), 24 CHG (88.9%) and 19 CHH (90.5%) methylation sites were demethylated (termed THG and THH, respectively), suggesting that long-term cold treatment caused universal demethylation of the *HbICE1* promoter (Fig. 5a). Notably, the CHG methylation site at the MBS (MYB binding site) *cis*-element was demethylated in the 2-CT sample (Additional file 1: Figure S3A), suggesting that demethylation of the *cis*-element alters the binding capacity and transcriptional activity of MYB. Surprisingly, almost all of the demethylated sites in 2-CT were switched to methylation in the 3-CT sample (Fig. 5a).

A similar pattern was observed in the *HbCBF2* promoter. The 318-bp *HbCBF2* promoter region contained *cis-*elements such as ABRE, ARE, CAAT, CAT, G-boxes, HSE, and TATA-boxes (Additional file 1: Table S3). The numbers of CGN, CHG, and CHH methylation sites were 8, 11, and 54, respectively. In the 1-Mock, 2-Mock, and 3-Mock control samples, no methylation sites underwent demethylation. Few demethylation sites were observed in the 1-CT sample (Fig. 5b). However, 48 THH (88.9%), 7 TGN (87.5%), and 11 THG (100%) demethylation sites were detected in the 2-CT sample. Many demethylation sites were located in TATA and ABRE boxes, allowing RNA polymerase and ABRE-binding transcription factors to bind the promoter and regulate the transcription of *HbCBF1* (Additional file 1: Figure S3B). As observed for *HbICE1*, most demethylation sites returned to a hypermethylation status after 3-CT (Fig. 5b).

The 287-bp fragment of the *HbMET* promoter contained many *cis*-elements; e.g. AT, CAAT-boxes, ERE, GARE, HSE, and P-boxes (Additional file 1: Table S3). The promoter sequence contained 44 methylation sites, including 3 CGN, 5 CHG, and 36 CHH sites. After the first cold treatment (1-CT), almost all methylation sites were demethylated, including 3 TGN (100%), 5 THG (100%), and 35 THH (97.2%) sites, whereas the methylation pattern of the 1-Mock, 2-Mock, and 3-Mock control samples was relatively unchanged. The hypomethylation status was maintained in the 2-CT and 3-CT samples (Fig. 5c), indicating that cold induced hypomethylation status of the *HbMET* promoter is more sustainable to cold treatment.

**Fig. 6:**
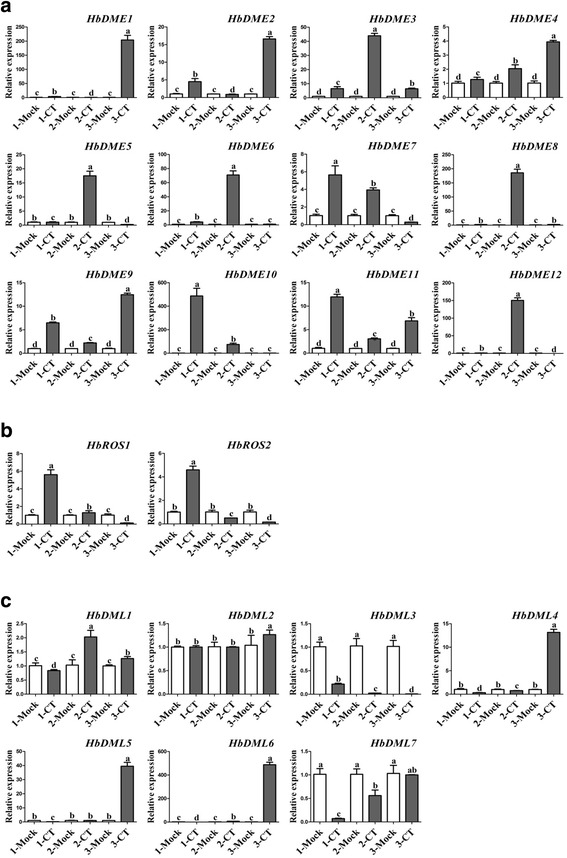
Long-term cold treatment regulated the expression of demethylation-related genes. Seedlings of *H. brasiliensis* were treated at 19 °C for 1 month (1-CT), transferred to 28 °C for recovery for 1 month, and then treated at 19 °C for 1 month (2-CT). After recovery for 1 month, the seedlings were again cold-treated for 1 month (3-CT). After each treatment, leaf samples were collected for qRT-PCR. (**a**) *HbDME*, (**b**) *HbROS*, and (**c**) *HbDML* genes. Values are presented as the mean standard error of three independent biological replicates. Different letters indicate significant differences (*P* < 0.05) according to one-way analysis of variance

Cold stress induced promoter hypomethylation and increased the expression of DNA methylation-associated genes, such as *HbMET* (Figs. 4 and 5). How can the increased DNA methyltransferase transcription and low promoter DNA methylation levels induced by cold treatment be explained? As described above, active removal of cytosine methylation is catalysed by DME, ROS, and DML, members of the DNA glycosylase family [27, 34, 35, 40]. In silico cloning identified 12 *HbDME*, 2 *HbROS*, and *7 HbDML* genes in the *H. brasiliensis* genome. qRT-PCR showed that these demethylation-associated genes, with the exception of *HbDML3* and *HbDML7*, were strongly induced by long-term cold treatment (Fig. 6). However, bisulphite sequencing revealed that none of these genes’ promoters were demethylated after long-term cold treatment (Additional file 1: Figure S4). These results suggest that cold induced the transcription, but did not alter the demethylation status of demethylation-associated genes.

### Methylation status of *HbICE1* and *HbMET* promoters according to geographical location and season

To investigate whether hypomethylation of *HbICE1* and *HbMET* could be induced by natural environmental conditions, we selected four rubber plantation locations—Sanya (N18°19′58.58′′; E109°27′35.35′′) and Wenchang (N19°37′37.29′′; E110°38′37.29′′), Hainan Province; and Xishuangbanna (N22°00′55.15′′; E100°46′13.36′′) and Puer (N22°47′45.75′′; E100°58′57.72′′), Yunnan Province. The four locations have mean temperatures ranging from 14.0 to 21.4 °C in winter and from 22.3 to 28.7 °C in summer (Table 1). Leaf samples of six cultivars from Puer, five cultivars from Xishuangbanna, four cultivars from Wenchang, and three cultivars from Sanya were collected from mature rubber trees in April and October 2016. Total DNA was isolated and treated with or without bisulphite. The promoter sequences of *HbMET* and *HbICE1* were amplified by PCR and sequenced. Methylation patterns were evaluated using CyMATE software.

**Fig. 7:**
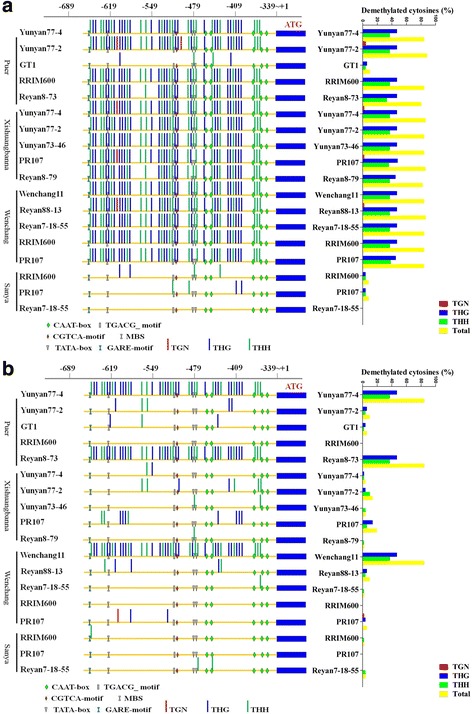
Effects of season and geographical location on the methylation status of *HbICE1* promoters. Leaves of six cultivars from Puer and five cultivars from Xishuangbanna, Yunnan Province; and from four cultivars from Wenchang and three cultivars from Sanya, Hainan Province were collected in April (**a**) and October (**b**). Total DNA was isolated and treated with or without bisulphite. The promoter sequence of *HbICE1* was amplified by PCR and sequenced. The methylation pattern was evaluated by CyMATE software. The percent of each demethylation type were indicated at the right side

Among 18 samples of 12 cultivars collected from the four locations, only PR107 had a single nucleotide polymorphism (SNP) in the 324-bp region of the *HbICE1* promoter; 14 samples (77.8%) collected in April showed evident hypomethylation in the analysed region of the *HbICE1* promoter (Fig. 7a). The demethylation patterns (number, type, and position) were almost identical to those in the long-term cold-treated sample (2-CT) in chamber condition (Fig. 5a), suggesting that demethylation is induced by low temperature. Interestingly, among the four samples that did not show hypomethylation, three (RRIM600, PR107, and Reyan 7–88-5) were collected from Sanya, the most southern city in China with the highest winter temperature (Fig. 7a). This DNA demethylation was not dependent on cultivar, because the cultivars collected in Sanya (RRIM600, PR107, and Reyan 7–88-5), were also collected in other locations, confirming that environmental temperature plays a key role in regulation of demethylation of the *HbICE1* promoter in the rubber tree. Another exception was the cultivar GT1, collected in Puer, the northernmost rubber plantation location in China, which showed hypermethylation of the *HbICE1* promoter, suggesting that other environmental factors might also influence epigenetic modification of the *HbICE1* promoter (Fig. 7a).

The *HbICE1* promoters of 15 samples (83.3%) collected in October, when rubber trees had undergone summer acclimation for several months, switched to a hypermethylation status (Fig. 7b). There was no correlation between methylation pattern and genotype. This confirmed that environmental temperature plays an important role in regulation of methylation of the *HbICE1* promoter. However, there were exceptions; e.g. two samples collected from Puer (Yunyan77–4 and Reyan77–2) and one sample collected in Wenchang (Wenchang 11), which retained a hypomethylation status (Fig. 7b), suggesting that environmental factors other than temperature affect the methylation status of the *HbICE1* promoter.

**Fig. 8:**
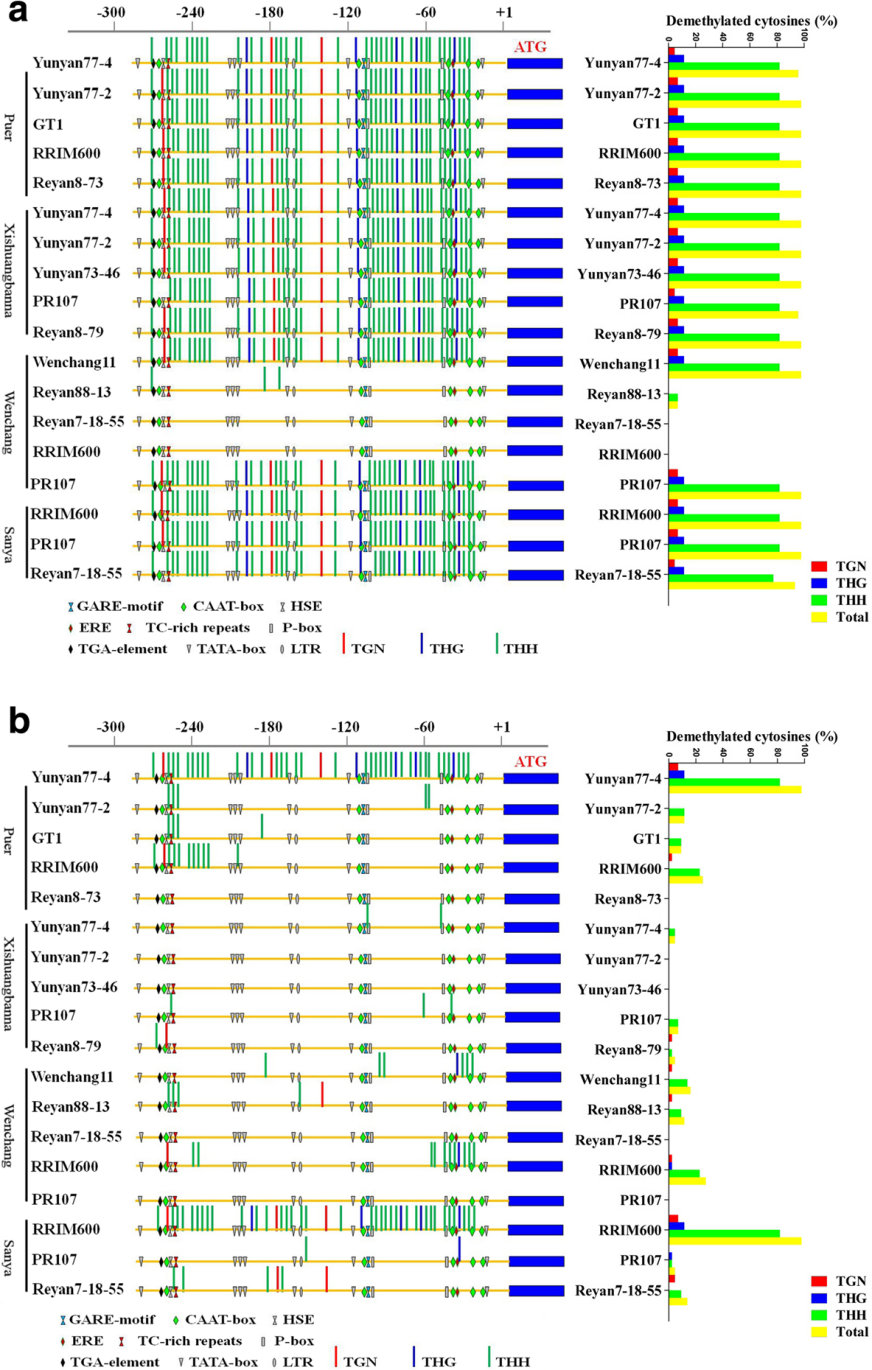
Effects of season and geographical location on the methylation status of *HbMET* promoters. Leaves of six cultivars from Puer and five cultivars from Xishuangbanna, Yunnan Province; and from four cultivars from Wenchang and three cultivars from Sanya, Hainan Province were collected in April (**a**) and October (**b**). Total DNA was isolated and treated with or without bisulphite. The promoter sequence of HbMET was amplified by PCR and sequenced. The methylation pattern was evaluated using CyMATE software. The percent of each demethylation type were indicated at the right side

A similar switch in methylation status was observed in the 287-bp fragment of the *HbMET* promoter. Among 18 samples of 12 cultivars, no SNP was found in the 287-bp region of the *HbMET* promoter. As expected, most samples collected in April after winter cold acclimation showed strong hypomethylation (Fig. 8a). Furthermore, the demethylation patterns (number, type, and position) were identical to those in long-term cold-treated samples in chamber condition (Fig. 5c). Unexpectedly, the *HbMET* promoter was hypermethylated in three cultivars collected from Wenchang, Hainan Province in winter, and so was affected by unknown environmental factors (Fig. 8a). As expected, the *HbMET* promoter of almost all samples (88.9%) collected in October switched to a hypermethylation status after summer acclimation (Fig. 8b).

### Gene expression patterns according to geographical location and season

**Fig. 9:**
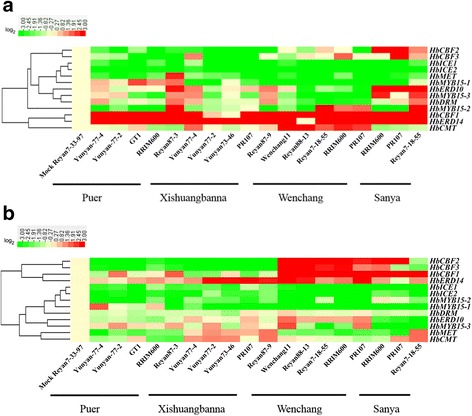
Effects of season and geographical location on gene expression patterns. Leaves of six cultivars from Puer and five cultivars from Xishuangbanna, Yunnan Province, and from four cultivars from Wenchang and three cultivars from Sanya, Hainan Province were collected in April (**a**) and October (**b**). Total RNA was isolated to produce cDNA. Gene expression patterns were analysed by qRT-PCR. The expression heatmap was generated using Java Treeview 4 [51]

To investigate the effects of geographical location and season on the expression and methylation status of *COR* methylation-related genes, we subjected the *HbICE1*, *HbICE2*, *HbCBF1*, *HbCBF2*, *HbCBF3*, *HbERD10*, *HbERD14*, *HbMYB15–1*, *HbMYB15–2*, *HbMYB15–3*, *HbMET*, *HbCMT*, and *HbDRM* genes to qRT-PCR analysis*. HbACT7b* was used as controls. Cluster analysis of transcriptional profiles was performed using CLUSTER [56], and a heatmap was generated by Java Treeview 4 [51]. Although these genes are regulated by short- or long-term cold treatment (Figs. 1, 2, 3, and 4), they did not specifically respond to temperature; therefore, other biotic/abiotic factors might alter their expression. The expression of several of these genes, such as *HbMYB15–2*, *HbCBF1*, *HbERD14*, and *HbCMT*, was significantly higher in the samples collected in April (Fig. 9a) than in October (Fig. 9b). This indicates a correlation between low temperature and expression of *COR* genes. No correlation with cultivar was observed (Fig. 9).

## Discussion

As a perennial tropical plant, *H. brasiliensis* is native to the Amazonian forests of Brazil. Because the cultivated clones were derived from a few original ‘Wickham base’ seedlings, they represent only a small part of the germplasm. The limited genetic variation of the current cultivars is insufficient to carry out breeding for genetic improvements in rubber production or biotic/abiotic tolerance. The vegetative model of propagation has further narrowed the genetic base. This hypothesis was supported by genome sequencing results. Few SNPs were observed among cultivars Reyan7–33-97, RRIM600, PR107, Wenchang11, Yunyan77–4, and Reyan8–79. The density of SNPs among all cultivars averages 2 per kilobase, and 95.1–95.6% occurred in non-coding regions. Furthermore, extremely low SNP regions (< 1 SNP kb), termed as ‘SNP deserts’, accounted for 42% of the *Hevea* genome [57], which is significantly higher than that reported in rice (8%) [58] or date palm (18%) [59]. However, different cultivars exhibit specific adaptation in their plantation areas. A number of clones, such as PB260, PR107, IRCA317, PB217, RRIM600, GT1, and Yunyan77–4, reportedly show different levels of cold tolerance [44, 45]. The successful extension of rubber tree cultivation to high latitudes is believed to benefit from long-term selective breeding of cold-resistant rubber tree clones [2, 60]. One puzzling incongruity is the remarkable divergence in quantitative and qualitative characters amongst the popular cultivars despite their similar genetic make-up. Furthermore, the long juvenile stage limits selective breeding. We therefore proposed that epigenetic modification might be responsible for the different characters of the cultivated clones.

The dominant stress on rubber trees cultivated in high-altitude areas is low temperature. Under stress conditions, survival strategies of seed plants include gene mutation [61], genome recombination [62], and epigenetic modification [63]. However, the survival strategies of vegetatively propagated rubber tree populations are limited. Uthup et al. first reported site-specific epigenetic modification induced by environmental factors in rubber trees [64]. However, how and which factors affect acclimation of the rubber tree to unfavourable conditions is unclear. In this work, we showed that cold treatment regulated the expression of *HbCBFs* and *HbICEs* genes (Fig. 1), which are involved in controlling the expression of *COR* genes to cope with cold stress (Fig. 2), as in Arabidopsis [33]. The differential expression patterns of *HbICE1* and *HbICE2*, and among three *HbCBF* genes, at 19 °C and 4 °C (Fig. 1), suggest that these homologous genes play different roles in the response to low temperature. Interestingly, cold treatment altered the expression patterns of DNA-methylation associated genes, such as *HbMET*, *HbCMT*, and *HbDRM* (Fig. 3), indicating that epigenetic DNA modifications are involved in cold acclimation in *H. brasiliensis*. Thus, *H. brasiliensis* seedlings were transferred to long-term cold treatment, and methylated CpG island screening showed different epigenetic modifications between control and cold-treated samples (Additional file 1: Figure S1). Bisulphite sequencing confirmed that the *HbICE1*, *HbCBF2*, and *HbMET* promoters became hypomethylated (Fig. 5), which is in agreement with induction of gene expression (Fig. 4). Many demethylation sites occurred at *cis* acting elements recognized by cold related transcriptional factors, for example, the MBS (MYB binding site) *cis*-element in *HbICE1* promoter (Additional file 1: Figure S3A). Cold treatment induced hypomethylation of retrotransposon areas of the genome of tobacco [65]. Our findings reveal that cold treatment induced hypomethylation of *ICE-CBF* promoter regions in *H. brasiliensis*, which is directly associated with acquisition of cold tolerance*.*

Other evidence that epigenetic modification is involved in cold acclimation of *H. brasiliensis* was identification of conversion of hypermethylation to hypomethylation of the *HbMET* promoter by long-term cold treatment (Fig. 5c). However, MET mediates establishment and maintenance of cytosine methylation. Why did cold stress induce *HbMET* transcription and hypomethylation in the promoters of *HbICE1*, *HbCBF2*, and *HbMET*? To resolve this issue, we analysed the expression of genes associated with removal of cytosine methylation. In total, 12 *HbDMEs*, 2 *HbROSs*, and *7 HbDMLs* genes in the *H. brasiliensis* genome were identified by an in silico procedure. Among 19 demethylation-associated genes, 17 were strongly induced by long-term cold treatment (Fig. 6). Therefore, cold treatment induced hypomethylation was reasonable, although how the demethylation-associated genes and HbMET function synergistically to recognise and modify their target genes is unclear. Additionally, bisulphite sequencing revealed that no conversion of methylation status occurred in the promoters of demethylation-associated genes after cold treatment (Additional file 1: Figure S4), indicating that initiation of the transcription but not the demethylation of the demethylation-associated genes is a prerequisite for DNA demethylation.

We further investigated whether cold-induced DNA demethylation plays a role in the cold acclimation of rubber trees in a natural environment. Rubber plantations in four locations were selected, including the southernmost and northernmost rubber plantation sites in China (Table 1). Samples were collected in April and in October, respectively. Although different cultivars were planted in the four locations, the cultivars collected overlapped. Although methylation status is regulated by multiple environmental factors, the demethylation of CpG islands in the *HbICE1* promoter was highly correlated with low temperature. Of the samples collected in April, 77.8% showed hypomethylation (Fig. 7). Of the four samples that were not hypomethylated, three were collected from Sanya, which has a relatively high winter temperature (Table 1). The demethylation patterns (number, type, and position) were almost identical, as was the case in samples subjected to cold treatment in chamber (Fig. 5a). After summer acclimation, hypomethylation was switched to hypermethylation, suggesting that DNA demethylation is caused by low temperature, not other environmental factors. A similar pattern was observed in the *HbMET* promoter. The *HbMET* promoter was hypomethylated in most samples collected in April but hypermethylated in October (Fig. 8), suggesting that the hypomethylation status of *HbMET* target genes is maintained until April, although which genes are targets of *HbMET* is unknown. Although *COR* genes are regulated not only by cold but also by a number of biotic and abiotic stresses, the number of *COR* genes expressed at a high level was greater in samples collected in April than in October, indicating a correlation between low temperature and *COR* gene expression (Fig. 9).

Notably, although the *HbICE1* and *HbMET* promoters were hypomethylated in most samples subjected to cold stress, there were several exceptions. For example, three samples collected in Wenchang in April showed hypermethylation and one sample (RRIM600) collected in Sanya showed hypomethylation of the *HbMET* promoter in October (Fig. 8). Similar exceptions were observed in the *HbICE1* promoter (Fig. 7). As we know, neither *HbMET* nor *HbICE1* are specifically responsive to cold stress and their expressions could be affected by multiple biotic/abiotic environmental factors such as temperature, salt, light, and osmotic stress. Each sample collected from different locations represents an individual stress combination of different environmental factors. The epigenetic modifications, e.g. DNA methylation status in *HbMET* and *HbICE1*, are coordinated by these factors. Although a common factor was cold stress for all samples, it is not difficult to understand the exceptions observed in this work, which representing different stress combinations.

Besides the temperature, altitude (height above sea level) is a major difference among Sanya, Wenchang, Xishuangbanna, and Puer. Different altitudes mean different environmental conditions, including temperature, light density, humidity, and so on, among which temperature might play a much more important role in limiting expansion of rubber tree to suboptimal area. In order to rule out the effects of other factors, rubber samples were corrected in different seasons, besides different latitudes, the switch of methylation status showed a close correlation with temperature (Figs. 7 and 8). Cold treatment induced the expression of many cold responsive genes and DNA methylation-related genes such as *HbICE1*, *HbCBF2*, *HbMET*, and *HbDME* genes. The induction of gene expression was closely correlated with cold-induced DNA demethylation in promoter. This correlation was also observed in 19 samples of 12 different cultivars collected from different geographical locations and in different seasons, and little genetic diversity was observed among these samples, reflecting the fact that low environmental temperature-induced epigenetic modifications (e.g. DNA demethylation) might play an important role in cold acclimation of *H. brasiliensis.* The alteration of genomic methylation status was prevalently documented under abiotic stresses [66–68], but cold induced DNA demethylation was not widely reported. In contrast to rubber tree that was introduced in China, crofton weed (*Ageratina adenophora*) is a highly invasive alien plant that is continuously spreading across subtropical areas in China through cold tolerance evolution. Recent report showed that demethylation-upregulated transcription level of CBF pathway is responsible for this evolution [69]. Results shown in this work confirmed that cold induced demethylation-upregulated transcription of ICE-CBF might also play a key role in cold acclimation in rubber tree. Recent report revealed that *ROS1* promoter functions like a thermostat (i.e., methylstat) to sense DNA methylation levels and regulates DNA methylation by controlling *ROS1* expression [70]. Whether rubber tree shares such a mechanism requires further investigation.

## Conclusions

Cold treatment not only induced the expression of cold-related genes and DNA-methylation related genes such as *HbICE1*, *HbCBF2*, and *HbMET*, but also induced the DNA demethylation in their promoters. Cold treatment increased the transcriptional activities of demethylation-related genes (*HbDME*, *HbROS*, and *HbDML*), but did not alter the promoter methylation status. The DNA methylation status of the *HbICE1* and *HbMET* promoters was closely related with the environmental temperature. Hypomethylation of *HbICE1* and *HbMET* promoters induced by cold was highly correlated with the expression level of *COR* genes, but not with the genetic backgrounds of cultivars. Little genetic diversity was observed in the *HbICE1* and *HbMET* promoters in different cultivars. It could be proposed that cold-induced epigenetic modification is more important than genetic variation in cold tolerance of different hevea cultivars*.*

## Additional file

Additional file 1: **Figure S1.** Methylated CpG island screening. **Figure S2.** Conversion rate of bisulfite sequencing. **Figure S3.** Representative Sanger sequencing chromatograms of the bisulphite-treated DNA samples of long-term cold treatment. **Figure S4.** Graphical representation of methylation patterns of three gene promoters altered by long-term cold treatment. **Table S1.** List of the primers for Quantitative real-time PCR. **Table S2.** List of the primers for PCR amplification of genes promoters. **Table S3.** Cis-elements existing in promoters of *pHbICE1*, *pHbCBF2* and *pHbMET*. (DOCX 1845 kb)

## Abbreviations

CBFs: C-repeat-binding factors
CMT: chromomethylase
COR: cold-responsive
CRT/DRE: C-repeat/dehydration response elements
CT: cold treatment
DME: Demeter
DML: demeter-like
DNMT: DNA methyltransferase
DREB: dehydration-responsive element-binding factor
DRM: domains rearranged methyltransferase
HOS: high expression of osmotically responsive genes
ICE: Inducer of CBF expression
MET: methyltransferase
RDM: RNA-directed DNA methylation
ROS: repressor of silencing
SAM: S-adenosyl-L-methionine
