# Supplementary material for: Chilling-induced DNA Demethylation is associated with the cold tolerance of Hevea brasiliensis
Source: BMC Plant Biol. 2018 Apr 23;18:70. doi: 10.1186/s12870-018-1276-7 (PMC5913804; doi:10.1186/s12870-018-1276-7)
Supplement: Supplementary file 1 — Figure S1. Methylated CpG island screening. Figure S2. Conversion rate of bisulfite sequencing. Figure S3. Representative Sanger sequencing chromatograms of the bisulphite-treated DNA samples of long-term cold treatment. Figure S4. Graphical representation of methylation patterns of three gene promoters altered by long-term cold treatment. Table S1. List of the primers for Quantitative real-time PCR. Table S2. List of the primers for PCR amplification of genes promoters. Table S3. Cis-elements existing in promoters of pHbICE1, pHbCBF2 and pHbMET. (DOCX 1845 kb) [file 12870_2018_1276_MOESM1_ESM.docx]

**Additional file 1:**


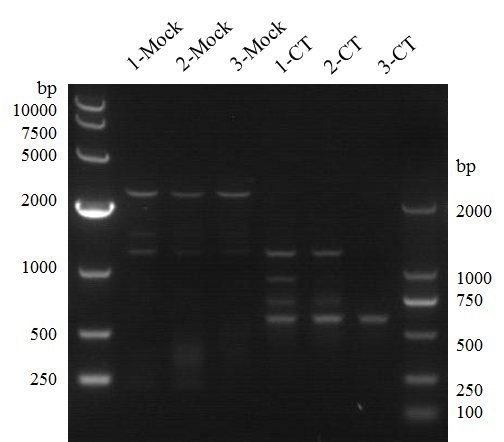


**Figure S1.** **Methylated CpG island screening.** Genomic DNA was isolated from leaves of *H. brasiliensis* and digested with *Sma*I and *Xma*I. Adapter oligonucleotides were ligated. The ligated fragments were amplified by PCR with an annealing temperature of 50°C. Differential display screening was performed using DNA fragments amplified by a second PCR using the initial PCR product as the template. Migration patterns were compared between the mock control and cold-treated (CT) samples. Three independent replicates were performed, one typical gel image was shown.

**
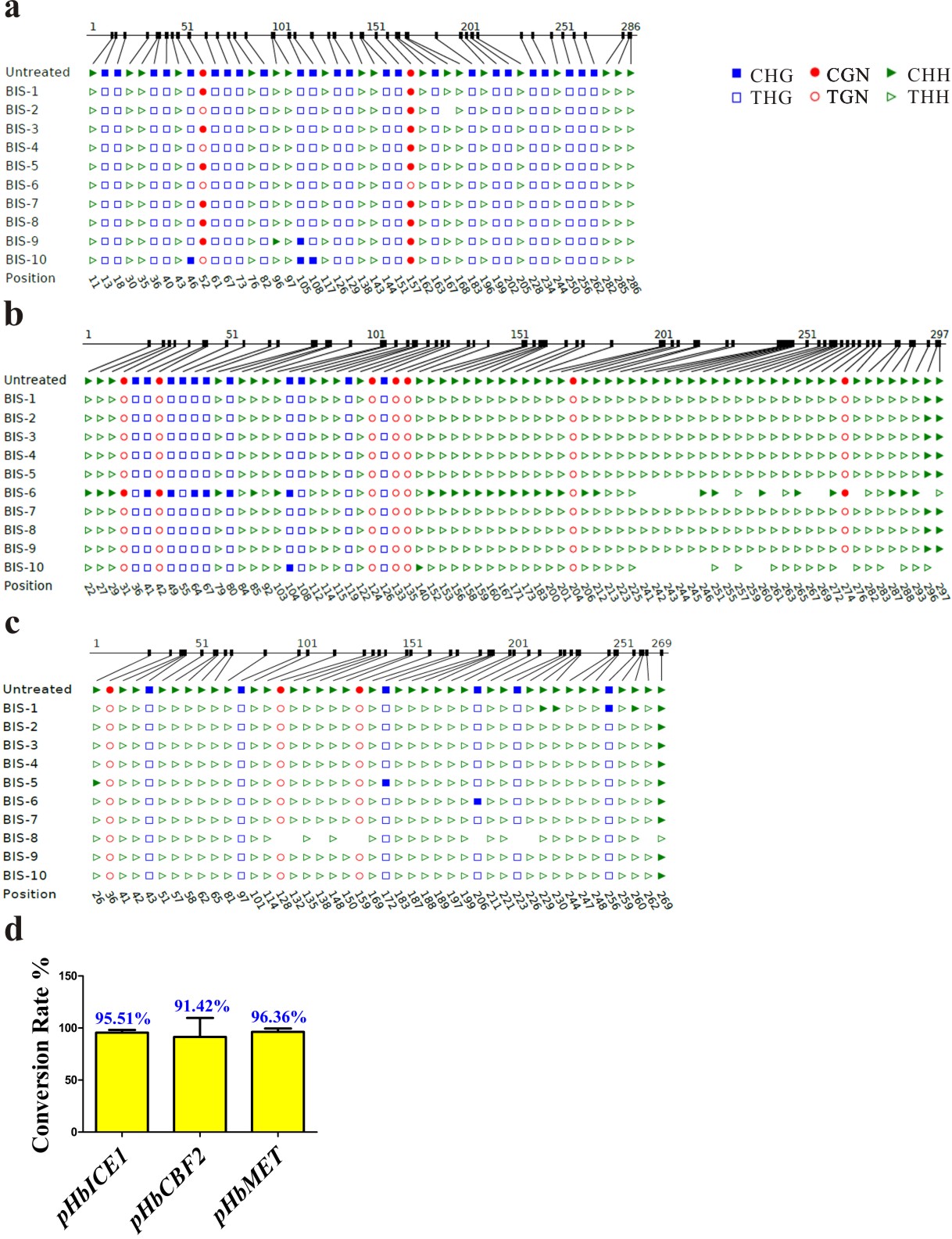
**

**Figure S2.** **Conversion rate of bisulfite sequencing**. PCR product of a 324-bp region of the *HICE1* promoter (**a**), a 318-bp region of the HbCBF2 promoter (**b**), and a 287-bp fragment of the HbMET promoter (**c**) were ligated into pMD-19T vector and transferred into DH5a *E. coli* strain, respectively. The plasmids were isolated for conversion rate of bisulfite sequencing. Each plasmid has 10 independent bisulfite treatments and sequencing. Methylation patterns were evaluated using CyMATE software (**a-c**). The conversion rate of bisulfite sequencing was calculated for promoter sequences of *HbICE1*, *HbCBF2* and *HbMET* (**d**).


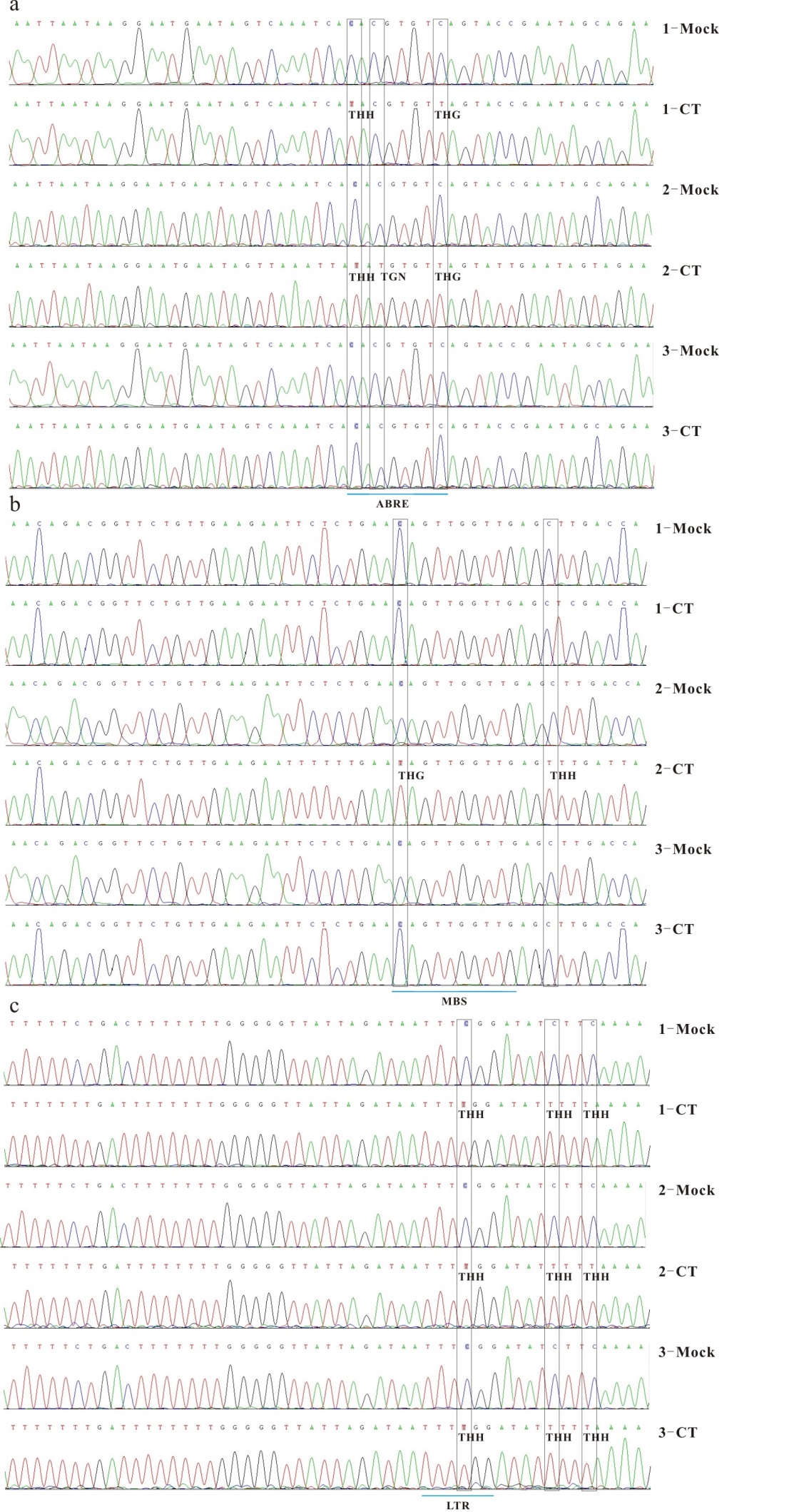


**Figure S3. Representative Sanger sequencing chromatograms of the bisulphite-treated DNA samples of long-term cold treatment.** Seedlings of *H. brasiliensis* were treated at 19°C for 1 month (1-CT), transferred to 28°C for recovery for 1 month, and then treated at 19°C for 1 month (2-CT). After recovery for 1 month, the seedlings were again cold-treated for 1 month (3-CT). Leaf samples were collected after each treatment. Total DNA was isolated and treated with bisulphite. Representative Sanger sequencing chromatograms of the bisulphite-treated DNA samples are shown at right, in which unmethylated sites are indicated by square frames. (**a**) A fragment of *HbICE1* promoter. (**b**) A fragment of *HbCBF2* promoter. (**c**) A fragment of the *HbMET* promoter.

**
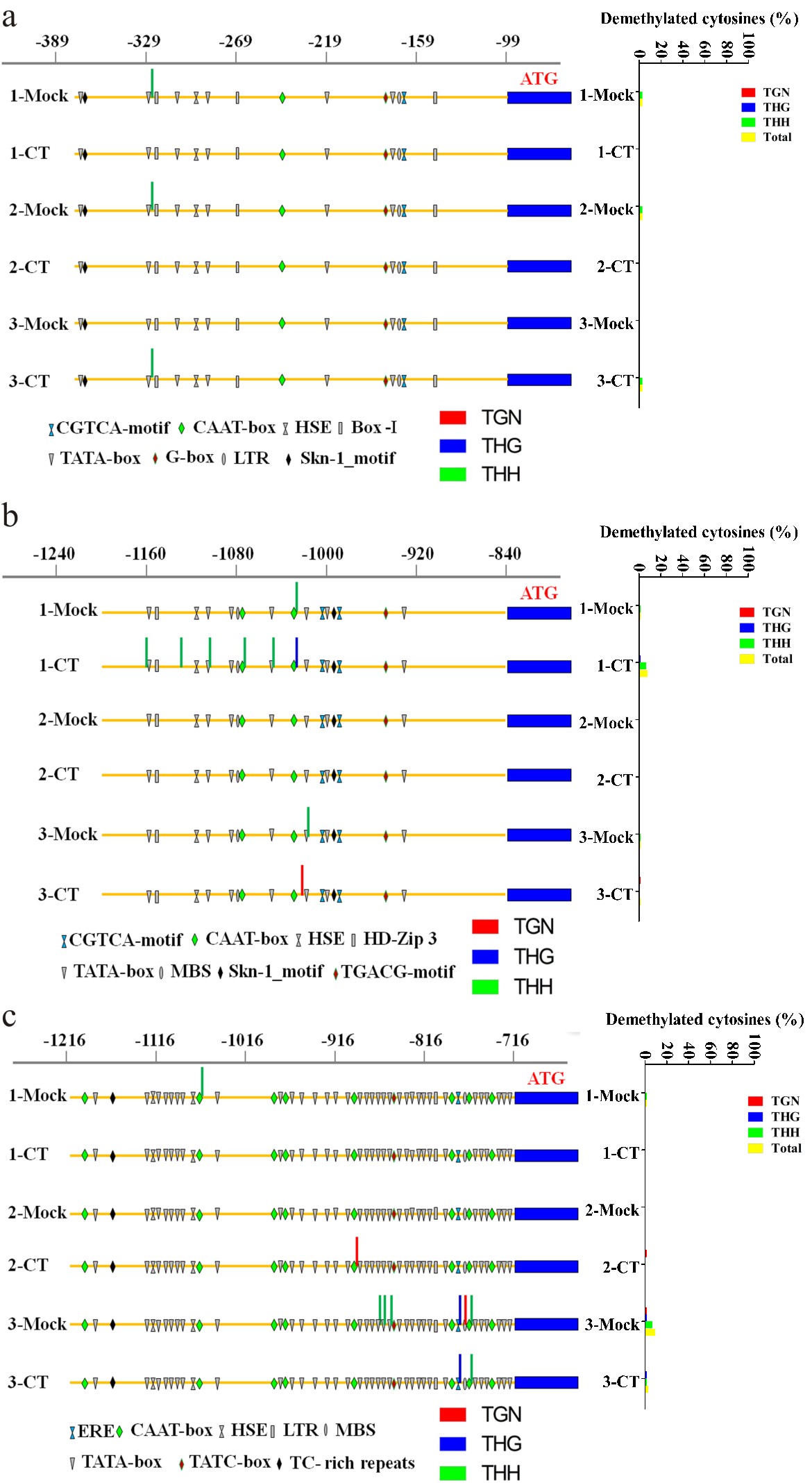
**

**Figure S4. Graphical representation of methylation patterns of three gene promoters altered by long-term cold treatment.** Seedlings of *H. brasiliensis* were treated at 19°C for 1 month (1-CT), transferred to 28°C for recovery for 1 month, then transferred to 19°C for 1 month (2-CT). After recovery for 1 month, the seedlings were again cold-treated for 1 month (3-CT). Leaf samples were collected after each treatment. Total DNA was isolated and treated with or without bisulphite. Methylation patterns were evaluated using CyMATE online software analysis. Probable TGN, THG, and THH demethylation sites were identified by the software and projected symbolically. Representative Sanger sequencing chromatograms of the bisulphite-treated DNA samples are shown at right, in which the unmethylated sites are indicated by square frames. (**a**) A 286-bp region of the *HDME3* promoter. (**b**) A 350-bp region of the *HbROS1* promoter. (**c**) A 492-bp fragment of the *HbROS2* promoter.

**Table S1. List of the primers for Quantitative real-time PCR.**

| Primer name Sequence (5’to 3’) | |
| --- | --- |
| HbRH8qRT-F  HbRH8qRT-R  Hb7aqRT-F  Hb7aqRT-R  Hb7bqRT-F  Hb7bqRT-R  HbICE1qRT-1F  HbICE1qRT-1R  HbICE2qRT-1F  HbICE2qRT-1R  HbCBF1qRT-5F  HbCBF1qRT-5R  HbCBF2qRT-4F  HbCBF2qRT-4R  HbCBF3qRT-3F  HbCBF3qRT-3R  HbERD10qRT-4F  HbERD10qRT-4R  HbERD14qRT-4F  HbERD14qRT-4R  HbMYB15-1qRT-5F  HbMYB15-1qRT-5R  HbMYB15-2qRT-5F  HbMYB15-2qRT-5R  HbMYB15-3qRT-1F  HbMYB15-3qRT-1R  HbMETqRT-F  HbMETqRT-R  HbCMTqRT-F  HbCMTqRT-R  HbDRMqRT-F  HbDRMqRT-R  HbDME1qRT-F  HbDME1qRT-R  HbDME2qRT-F  HbDME2qRT-R  HbDME3qRT-F  HbDME3qRT-R  HbDME4qRT-F  HbDME4qRT-R  HbDME5qRT-F  HbDME5qRT-R  HbDME6qRT-F  HbDME6qRT-R  HbDME7qRT-F  HbDME7qRT-R  HbDME8qRT-F  HbDME8qRT-R  HbDME9qRT-F  HbDME9qRT-R  HbDME10qRT-F  HbDME10qRT-R  HbDME11qRT-F  HbDME11qRT-R  HbDME12qRT-F  HbDME12qRT-R  HbROS1qRT-F  HbROS1qRT-R  HbROS2qRT-F  HbROS2qRT-R  HbDML1qRT-F  HbDML1qRT-R  HbDML2qRT-F  HbDML2qRT-R  HbDML3qRT-F  HbDML3qRT-R  HbDML4qRT-F  HbDML4qRT-R  HbDML5qRT-F  HbDML5qRT-R  HbDML6qRT-F  HbDML6qRT-R  HbDML7qRT-F  HbDML7qRT-R | TCACAGGGTTGGTAGATCAG  CCAAGCTCTTGCTCAATCC  CACCACCAGAGAGAAAGTACAG  GATGGACCAGACTCATCGTATTC  CAGTGTCTGGATAGGAGGATCTA  AAATGGACCGGACTCATCATAC  GTTCACCGCCAACTCTATT  CCCAATTTCTCCTCCATATCTC  GCACTCAAAGCCTACCTTATC  GAGGCATTTGTGGTTGTAGT  CAATGCCAGCTTCTTCATAAAC  CGTTCCAATTCAACCTCTCC  TGGCAGAAGGAATGCTATTG  CTGCTGCATCCGTTTCTT  CATGAAAGCCTCACGTACATA  CGAAGTCATCCCAGTTGATAC  TAAGTGTGTGGCTTGTAGTG  GTACAAAGCTTGCCCATAATC  TGAAGGAGAAGATCAAGGATAA  TCTCAACCTTCTCAATAGGAATAG  CAGGGATGGAAACCATCAAA  CATGAATTCACTTCAGGCTCTA  AAACCAATACCAGCCAATAGT  GTCCATCAGTTGTGTTTCATTAG  CCAGGGACCTTTGAGTTCTA  GCTGTCATTAAGAGTCCAATCC  CAGGGTTTCTCGGGAATGAATAG  GGGTCGGAAGTAGTCAACAAAG  CAAGGGCTGAACCTCACAATC  GCAATCAGGGAACCCTTGTAATC  GGTGGGAGTCCATGCAATAATC  CCAAGTCTAGAATGCGACAGAAAT  CTACACAGGCCTTAAAT  GACTGAGAATGTGGATAC  CAAATCCAAGCCCAGGTTCA  GGTGGTAATGGTGGTGTTGATAG  GGCCTCAGATAGATTTC  GAACCAAGGGACTTTAG  CTTACAGATGCTGGTTT  GAGATCCAATTTACAGAGA  CCACACAATGCTTAATG  AGAAGAACTGGCTTATC  GCAGCTTCTGTCATATC  GGAAGTCACAGACAAAC  CATCTTCACCCTTCTAC  CTCCAAGGGACTAATTC  GTCTGTGTGACCATAAA  TAGCTGCAAGTGTATTC  CCCTATCACTTCCTCTACCG  TGGAGAGAGTTTACGGACTTT  ACAGTAACTTCCTTTCC  GAATCAACACACTTTCC  GGGCACAAACTCTACTT  CTGCTTCACCTTCTTTC  CTGAAGTGATGCTACAA  TCCTCAAGAGAAGAGAG  CATACCTGTTGGAGATAC  GACTGGGAAAGCATTAT  CGAGGAGTCAAGTAATC  TGTTACAATTTGGCTTG  CACTCAACTGCATTTAC  ATCTGGTGGACCTATTA  CGGCAAAGATAGGTAAA  GGCTATGATTGGGTAAG  CACTGTTTAATGACACC  CATTTGCACGTCTTTTG  ATGATCCTTGCTCATAC  CACAGTTTGCCATATTC  CAAACCGTCCTAGTATC  CTATGGGAGGTAAGTTG  CCTAGTAAGATGGAAAGG  CAAGAACTAGGATCCAC  CTTTCCCTGTTGACATA  ACTGGGTACTCTTCTAA |

**Table S2. List of the primers for PCR amplification of genes promoters.**

| Primer name Sequence (5’to 3’) | |
| --- | --- |
| BpICE1-6F  BpICE1-6R  BpCBF1-1F  BpCBF1-1R  BpCBF2-4F  BpCBF2-4R  BpCBF3-2F  BpCBF3-2R  BpMET-4F  BpMET-4R  BpDME3-F  BpDME3-R  BpROS1-F  BpROS1-R  BpROS2-F  BpROS2-R | AACAGACGGTTCTGTTGAAGAATT  TTCAATGCAAGCAGCTAAACCATT  AGTGATAGAAACGGAAAAGAAAAAA  AAAACATCCATAGTAGAGAGGGAAA  AATTAATAAGGAATGAATAGT  CATTTCTTAAAAAATTATAAGGTT  TAGGAAATTGTTTAATGACTA  TACTTTAAATTTTGCCAATAA  ATATGTTTTTGATAGGATAATTATG  TCCTATTACCAAATACAAGTCCAAT  GATCAGTTTTAGTCATAAGAATACTA  CAACTTAAAGTACTTCAAACCGGTTC  CGTTAATTATTTTCTTCATGATCTTG  GCAGTACTGCGAAAGGCTTTATGCCT  CTATTAATCAATAATGCTATACGCC  GCCGTCTTTGTCGTTATTTGTACATC |

**Table S3. Cis- elements existing in promoters of *pHbICE1*, *pHbCBF2* and *pHbMET*.**

| Name | Core sequence | Component function | pHbICE1 (324bp) | pHbCBF2 (318bp) | pHbMET (287bp) |
| --- | --- | --- | --- | --- | --- |
| AT1-motif | AATTATTTTTTATT | Part of a light responsive module |  |  | +65(14)~79 |
| BRE | CACGTG/  CGCACGTGTC | Abscisic acid responsiveness |  | +26(10)~36; +28(6)~34 |  |
| ACE | GACACGTATG | Light responsiveness |  | -26(10)~36 |  |
| ARE | TGGTTT | Anaerobic induction | +276(6)~282; +302(6)~308 | -279(6)~285 |  |
| ATCC-motif | CAATCCTC | Part of a conserved DNA module involved in light responsiveness | -282(8)~290 |  |  |
| Box I | TTTCAAA | Light responsive element |  |  | -231(7)~238 |
| Box 4 | ATTAAT | Part of a conserved DNA module involved in light responsiveness |  | +1(6)~7 |  |
| Box-W1 | TTGACC | Fungal elicitor responsive element | +44(6)~50 |  |  |
| CAAT-box | CAAAT/CAATT/  CAAT/CCAAT/AGCTCAATTTCA/  TGCCAAC | Common cis-acting element in promoter and enhancer regions | -219(5)~224; +299(4)~303; -286(4)~290; -318(4)~322; -224(5)~229; +298(5)~303 | +21(5)~26; +282(5)~287; +84(4)~88; +283(4)~287; +83(5)~88; +149(7)~156 | +42(5)~47; -236(5)~241; -164(4)~168; -262(5)~267; -163(5)~168; -237(4)~241; -233(11)~244; -273(5)~278 |
| CGTCA-motif | CGTCA | MeJA-responsiveness | -167(5)~172 |  |  |
| ERE | ATTTCAAA | Ethylene-responsive element |  |  | -231(8)~239 |
| GARE-motif | TCTGTTG | Gibberellin-responsive element | +10(7)~17 |  | +170(7)~177 |
| GATA-motif/GTGGC-motif | GATAGGA/  GATTCTGTGGC | Part of a light responsive element | +273(11)~284 |  | +10(7)~17 |
| G-box | CACGTG | Light responsiveness |  | +28(6)~34 |  |
| MBS | CAACTG | MYB binding site involved in drought-inducibility | -31(6)~37 |  |  |
| HSE | AGAAAATTCG | Heat stress responsiveness |  |  | -42(10)~52 |
| LTR | CCGAAA | Low-temperature responsiveness |  |  | -124(6)~130 |
| O2-site | GATGATGTGG | Zein metabolism regulation | +237(10)~247 |  |  |
| P-box | CCTTTTG/  CCTTTTG | Gibberellin-responsive element |  |  | +187(7)~194;+228(7)~235 |
| Skn-1_motif | GTCAT | Endosperm expression | -152(5)~157 |  |  |
| Sp1 | CC(G/A)CCC | Light responsive element |  | +241(5)~246 |  |
| TATA-box | CcTATAAAaa/  TATA/ TAATA/TTTTA/TATAA/TATAAA/  ATATAA/TATAAAtc | Core promoter element around -30 of transcription start | -187(5)~192; -190(5)~195 | -187(5)~192; -190(5)~195 | -6(9)~15; +82(4)~86; -75(5)~80; -160(4)~164; +72(5)~77; -114(5)~119; -81(5)~86; +279(5)~284 |
| TGACG-motif | TGACG | MeJA-responsiveness | +167(5)~172 |  |  |
| TC-rich repeats | ATTTTCTTCA/ GTTTTCTTAC | defense and stress responsiveness |  | -140(10)~150 | +45(10)~55 |
